# Duty on Quinine

**Published:** 1879-11-20

**Authors:** 


### *DUTY ON QUININE.*

Upon the subject of the removal of the duty on the sulphate of quinine, recommended by the American Medical Association and adopted by the last Congress, this journal has not expressed itself; nor are we prepared now to say that the action taken on the matter was wise or judicious.

That the sulphate of quinine is somewhat cheaper is true, yet there have been times before the removal of the duty, when it was quite as low as at present. We suppose, of course, that the competition resulting from the influx of foreign preparations will have the effect of cheapening them, but this advantage may be counterbalanced by the introduction of adulterated and unreliable preparations. Already complaints are coming up that there are spurious articles in the market, and that double and treble the amount of quinine is required to produce the desired impression as formerly, and it is stated that there are now not less than fourteen different preparations of the article on the market.

Upon general principles it would seem that the removal of the tariff upon an article so universally demanded by the wants of humanity was right and proper, and it was natural to sympathize with this view of the subject, but we were not among those who sought to prejudice the minds of the public by raising the cry of monopoly and greed as against such time-honored establishments as those of Powers & Weightman, Warner & Co., Rosengarten and others, who had for long years devoted so much capital and skill in the manufacture of quinine and its preparations, and who had so faithfully and well supplied the market with articles so uniformly *pure* and reliable as to give character and precedence to American preparations over those of all other countries on the globe. We felt in our hearts a true American pride in this success of our manufacturers, and that so far from abuse they were entitled rather to the thanks and gratitude of the American people.

But it is not our object here to eulogize any particular parties or establishments, but simply to give impartial expression of our individual views.

The importance of pure and reliable medicines we have frequently discussed; it rises in our estimation above all mere personal or private considerations—even above that of cheapness or economy. If we have to use impure or spurious drugs, our patients must suffer, our reputation as practitioners go down, and the profession at large lose cast and usefulness in public estimation.

This much we say, not in advocacy of the tariff or against it, but as favoring a careful watching and consideration of the action taken, and to reiterate our often expressed views of the great importance of protecting the public and the profession against the manufacture of impure drugs.

